# Spatial and non-spatial aspects of neglect

**DOI:** 10.3389/fnhum.2013.00025

**Published:** 2013-02-05

**Authors:** Konstantinos Priftis, Mario Bonato, Marco Zorzi, Carlo Umiltà

**Affiliations:** ^1^Department of General Psychology, University of PadovaPadova, Italy; ^2^Laboratory of Neuropsychology, IRCCS San Camillo HospitalLido-Venice, Italy

Deficits of contralesional space awareness (neglect and extinction) often follow right hemisphere damage and are typically attributed to the disruption of neurocognitive mechanisms subserving orienting of attention in space (Driver and Vuilleumier, 2001). Neglect affects awareness of contralesional stimuli, whereas extinction affects contralesional awareness only when competing stimuli are presented in the ipsilesional space. The difference between neglect and extinction contributes to the complexity of the disorders of contralesional spatial processing, in which the heterogeneity of symptoms can be hardly reconciled with the impairment of a single underlying mechanism. A widely accepted theory (Posner et al., 1984) maintains that neglect and extinction are caused by a deficit in disengaging spatial attention from ipsilesional stimuli. This theory is based on the observation that patients with parietal brain damage are particularly slow to detect a target presented in the contralesional visual field when it is preceded by a spatial cue that directs attention to the ipsilesional visual field. Posner et al., therefore, suggested that parietal damage produces a bias toward the ipsilesional hemispace, so that spatial attention is pathologically stuck to the stimuli shown there (i.e., hyperattention). Because of this bias, contralesional stimuli would remain undetected because patients' spatial attention is prevented from disengaging from ipsilesional stimuli. Another hypothesis adds a non-spatial aspect to the explanation of extinction (Desimone and Duncan, 1995). The idea is that, because attentional resources are limited, the neural representations of the stimuli have to compete for these limited resources. In brain-damaged patients, this competition would be biased because of their unilateral lesion. As a consequence, the contralesional stimuli lose the competition with the ipsilesional stimuli for attracting attention. The hypotheses that non-spatial attentional (or processing) resources are limited and that non-spatial and spatial components interact in neglect and extinction are helpful in order to explain these complex phenomena (for reviews see Husain and Rorden, 2003; Bonato, 2012). For example, it has been shown that increased attentional demands, generated by a concurrent task, can impair contralesional space awareness in brain-damaged patients (Robertson and Frasca, 1992; Bonato et al., 2010, 2012).

The studies collected in the present Research Topic cover both spatial and non-spatial aspects of neglect and extinction. With respect to the anatomical basis of these disorders two studies use a meta-analytic approach based on anatomical likelihood estimation to investigate the heterogeneous nature of the neuroanatomical underpinnings of neglect. Molenberghs et al. (2012; see commentary by Bartolomeo, 2012) found specific anatomical clusters for distinct neglect subtypes (e.g., personal vs. extra-personal neglect). Chechlacz et al. (2012) focuses on the dissociation between egocentric and allocentric signs of neglect. Both studies suggest that different forms of neglect are linked to both distinct and common lesion patterns involving gray and white matter. Two review articles (Bartolomeo et al., 2012; Bonato, 2012) draw a picture of the rather complex interactions between attentional networks devoted to attentional orienting and highlight the role of non-specific attentional resources in compensating contralesional biases given that neglect clearly emerges on computer-based presentation of transient targets. Two studies (Dukewich et al., 2012; Fellrath et al., 2012) investigate visuospatial attention asymmetries in the processing of brief visual targets. Fellrath et al. (2012), using a preview paradigm, show a serial search strategy in the left hemifield of neglect patients, as opposed to the pop-out effect characterizing healthy controls. Dukewich et al. (2012) compare temporal order judgments and speed of detection in a spatial cueing task; they highlight the lack of correlation between the two tasks in terms of disengage deficit. Yamanashi Leib et al. (2012) investigate the extraction of summary statistics (mean object size) in the left and right hemifield of patients with mild neglect. One long-standing issue in neglect is the difference between premotor and attentional disorders. Loetscher et al. (2012) propose a neat method to disambiguate output-related components from input-related components by asking patients to perform line bisection first and then to judge their own performance in a landmark task. Two studies explore the boundaries between rehabilitation procedures and the study of body schema, which could be distorted when neglect extends to personal space. Reinhart et al. (2012) show that limb activation (but not alertness cueing) ameliorates the judgment on the orientation of visually presented hands. Bolognini et al. (2012) show that bisection of real body parts dissociate from bisection of fake body parts and that both can be ameliorated by means of prismatic adaptation. Body schema, however, has several dynamic properties and can be modulated by different inputs. The interaction between body schema and vision has been highlighted in the study by Sambo et al. (2012), who show that bringing the patient's left hand in the right hemispace modulates both reaction times and early somatosensorial evoked potentials to tactile stimuli, particularly when the hand is in the patient's sight. An intriguing perspective comes from the study of Maravita et al. (2012), who show that tactile extinction decreases following hypnotic suggestion. This is the first study demonstrating that hypnosis can be useful not only to induce but also to ameliorate a neuropsychological disorder, in this case contralesional awareness deficits emerging when competitive stimuli are presented ipsilesionally (i.e., extinction). The variety of studies reported in the present Research Topic confirms that deficits of contralesional space awareness can substantiate into a variety of forms. Advanced approaches, as those presented in the present Research Topic, go well beyond the current clinical and experimental standards, and seem to be the key to better understand the nature of contralesional hemispace awareness deficits.
